# Editorial: Adrenal related hypertension: from bench to bedside

**DOI:** 10.3389/fendo.2023.1258052

**Published:** 2023-08-22

**Authors:** Norlela Sukor, Elena Aisha Binti Azizan

**Affiliations:** Department of Medicine, Faculty of Medicine, The National University of Malaysia, Universiti Kebangsaan Malaysia (UKM), Kuala Lumpur, Malaysia

**Keywords:** endocrine hypertension, adrenal, aldosterone, primary aldosteronism, hypertension, secondary hypertension

This Research Topic brings together 6 original research and review articles that elucidates the clinical presentations and molecular mechanisms that underly common adrenal-related endocrine hypertension. In addition, the current diagnostic process during screening and confirmatory tests and their limitations are discussed. Five of the research articles focus on primary aldosteronism (PA), a common cause of adrenal related endocrine hypertension, characterized by excessive aldosterone secretion from the adrenal glands, and in some cases hypokalemia (1, 2). The two main forms of PA are aldosterone-producing adenomas (APA) and bilateral idiopathic hyperaldosteronism (IHA). PA is a major public health concern as it is associated with increased cardiovascular events compared to essential hypertension due to aldosterone-induced cardiovascular damage (3, 4). Treatment options include adrenalectomy or the use of aldosterone receptor blockers which has been found to reverse the cardiovascular damages (5, 6).

A study by Lee et al. focuses on the impact of KCNJ5 mutations, frequently observed in APAs, on atherosclerotic parameters. The study included 179 APA patients who underwent adrenalectomy. Patients with mutation were noted to have increased aortic wall thickness but lower aortic calcification scores. Following adrenalectomy, mutation carriers exhibited less progression of aortic wall thickness compared to non-carriers. These findings suggest that KCNJ5 mutations are associated with atherosclerotic changes in PA patients.

Another study by Gomez-Sanchez et al. presents a case study of a black teenage patient with a KCNJ5 mutant APA that presented with drug-resistant hypertension and persistent hypokalemia. APA is rare in pediatric patients, and black patients have a higher susceptibility to mineralocorticoid-induced hypertensive effects. The patient’s blood pressure and kidney function improved after unilateral adrenalectomy, emphasizing the importance of early diagnosis and tailored treatments for higher-risk populations. Genetic analysis of excised adrenal revealed a novel pI157S somatic KCNJ5 mutation.

Using CYP11B2 immunohistochemistry-guided targeted next-generation sequencing, Tseng et al. also identified a novel somatic mutation, V1937M CACNA1H, in a patient with sporadic PA. CACNA1H, which encodes the voltage-dependent calcium channel Cav3.2, have been rarely found in PA due to sequencing challenges. Functional analysis revealed that the mutant gene increased aldosterone secretion and levels of aldosterone synthase. This expands our understanding of the genetic basis of PA and its clinical and pathological features of different PA subtypes.

The Research Topic also includes a systematic review and meta-analysis of the diagnostic value of liquid chromatography-tandem mass spectrometry (LC-MS/MS) method for APA in patients with hypertension (Hua et al.). LC-MS/MS is considered a more reliable and specific method for measuring plasma aldosterone concentration. It detects substances based on mass-to-charge ratio, offering high sensitivity and specificity. Some specialized laboratories have adopted LC-MS/MS as the “gold standard” for hormone detection. However, the diagnostic value of LC-MS/MS for PA is debated due to small sample sizes, lack of standardized protocols, and clinical heterogeneity. Herein, Hua et al.‘s meta-analysis included 12 studies with 4,191 participants, analyzing sensitivity, specificity, and diagnostic odds ratios. Hua et al. demonstrated that LC-MS/MS had good overall accuracy, sensitivity, and specificity, suggesting its diagnostic value. However, larger studies with standardized protocols are needed to establish consensus.

Similarly, Shi et al. have developed a machine learning-based prediction model to diagnose the subtypes of PA. Discriminating between unilateral PA and bilateral PA is crucial for determining appropriate treatment strategies. The current gold standard for diagnosis, adrenal venous sampling, has limitations, necessitating improved methods. The study used data from the public database “Dryad” and included ten parameters to develop the prediction model using machine learning technology. The model was then validated in an external dataset. The prediction model incorporated variables such as age, sex, blood pressure, aldosterone to renin ratio, serum potassium, and response to certain tests. The model achieved high accuracy and could differentiate between unilateral and bilateral PA without the need for CT imaging, reducing reliance on invasive procedures. This model has the potential to improve clinical decision-making and patient management. While the results are promising, its utility should be determined in future prospective trials.

Last but not least, the article by Asla et al. sheds light on a rarer but still important cause of endocrine hypertension. The authors showcased a patient who presented with acute onset of resistant hypertension and severe hypokalemia whereby the diagnosis of 11- deoxycorticosterone producing adrenal hyperplasia was made through biochemical, radiological and histological findings. This case report is followed by systematic review of the literature on this rare condition. The article provides valuable cognizance into the diagnosis and management of this rare endocrine cause of hypertension. Asla et al. emphasized the importance of considering this rare condition in the differential diagnosis of resistant hypertension especially in a setting of low renin and low normal aldosterone levels. In general, this article adds to the gap in the literature as it provides important insights into the scarcity of knowledge in this area. It also highlights the need for prompt diagnosis and management in order to provide better care and avoid potential complications.

Overall, these articles contribute to our understanding of endocrine hypertension’s clinical manifestations, molecular mechanisms, diagnostic approaches, and potential treatment strategies. Further research is needed to enhance our knowledge and improve patient outcomes in this field.

## Author contributions

NS: Writing – original draft, Writing – review & editing. EA: Writing – original draft, Writing – review & editing.
